# Calcium Metal Batteries with Long Cycle Life Using a Hydride‐Based Electrolyte and Copper Sulfide Electrode

**DOI:** 10.1002/advs.202301178

**Published:** 2023-05-19

**Authors:** Kazuaki Kisu, Rana Mohtadi, Shin‐ichi Orimo

**Affiliations:** ^1^ Institute for Materials Research (IMR) Tohoku University Katahira 2‐1‐1, Aoba‐ku Sendai 980‐8577 Japan; ^2^ Materials Research Department Toyota Research Institute of North America Ann Arbor MI 48105 USA; ^3^ Advanced Institute for Materials Research (WPI‐AIMR) Tohoku University Katahira 2‐1‐1, Aoba‐ku Sendai 980‐8577 Japan

**Keywords:** calcium metal anodes, copper sulfide, hydride‐based electrolytes, long‐term cycling, rechargeable calcium batteries

## Abstract

As potential alternatives to Li‐ion batteries, rechargeable Ca metal batteries offer advantageous features such as high energy density, cost‐effectiveness, and natural elemental abundance. However, challenges, such as Ca metal passivation by electrolytes and a lack of cathode materials with efficient Ca^2+^ storage capabilities, impede the development of practical Ca metal batteries. To overcome these limitations, the applicability of a CuS cathode in Ca metal batteries and its electrochemical properties are verified herein. Ex situ spectroscopy and electron microscopy results show that a CuS cathode comprising nanoparticles that are well dispersed in a high‐surface‐area carbon matrix can serve as an effective cathode for Ca^2+^ storage via the conversion reaction. This optimally functioning cathode is coupled with a tailored, weakly coordinating monocarborane‐anion electrolyte, namely, Ca(CB_11_H_12_)_2_ in 1,2‐dimethoxyethane/tetrahydrofuran, which enables reversible Ca plating/stripping at room temperature. The combination affords a Ca metal battery with a long cycle life of over 500 cycles and capacity retention of 92% based on the capacity of the 10th cycle. This study confirms the feasibility of the long‐term operation of Ca metal anodes and can expedite the development of Ca metal batteries.

## Graphical abstract

A long‐cycle‐life calcium metal battery is developed using a CuS/C composite and a tailored monocarborane electrolyte solution of Ca(CB_11_H_12_)_2_ in dimethoxyethane/tetrahydrofuran, which enables reversible Ca plating/stripping at room temperature. The combination affords a calcium metal battery with a long cycle life of over 500 cycles and capacity retention of 92% based on the capacity of the 10th cycle.

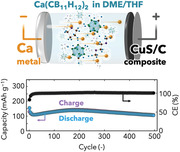

## 1 Introduction

The accelerating demand for electric vehicles and grid‐scale energy storage systems has motivated the exploration of next‐generation energy storage devices, which are anticipated to have higher energy densities and lower costs than existing lithium‐ion batteries (LIBs).^[^ ^1^ ^]^ In particular, divalent‐metal‐based batteries are receiving considerable attention as post‐LIB technologies owing to their higher energy densities, cost‐effectiveness, and safety/stability features.^[^ ^2^ ^]^ Additionally, divalent‐ion storage involves double‐electron transfer per ion in the redox reaction, potentially leading to higher energy densities than those achieved using monovalent ions. Consequently, rechargeable multivalent‐metal‐based batteries featuring Mg^2+^, Zn^2+^, and Ca^2+^ ions have attracted significant attention over the past decade, and research on them has made substantial progress in terms of achieving superior battery performance.^[^ ^3^ ^]^

Batteries with Ca metal anodes appear competitive in terms of realizing high energy densities, given the high volumetric/gravimetric capacities (2072 mAh cm^−3^/1337 mAh g^−1^) and low reduction potential (−2.87 V vs the standard hydrogen electrode [SHE]) of the Ca metal.^[^ ^4^ ^]^ Additionally, as the charge density of Ca^2+^ (0.49 e Å^−3^) is lower than that of other divalent ions, such as Mg^2+^ and Zn^2+^ (1.28 and 1.18 e Å^−3^, respectively), the softness of Ca^2+^ ions induces a tendency to form more covalent bonds with host anions, which may accelerate ion transport and diffusion in electrolytes and cathode materials.^[^ ^4^, ^5^ ^]^ These advantageous characteristics of Ca—in addition to its abundance in the crust and environmental compatibility—highlight the significance of Ca metal batteries as a promising post‐LIB candidate.^[^ ^6^ ^]^ However, the research and development of Ca metal batteries involve various challenges including the lack of an efficient electrolyte and a practical cathode that promotes the reversible Ca^2+^ storage reaction.^[^ ^7^ ^]^

Considerable research efforts have recently been devoted to cathode materials while being influenced by the development of electrolytes.^[^ ^8^ ^]^ As electrolytes, salts, such as Ca(BF_4_)_2_, Ca(PF_6_)_2_, and Ca(TFSI)_2_, in carbonate solvents—which are similar to the electrolyte systems of LIBs—have been utilized to evaluate potential cathodes exhibiting Ca^2+^ storage attributes.^[^ ^9^ ^]^ However, these electrolytes are generally less conducive to Ca metal plating/stripping at room temperature,^[^ ^10^ ^]^ with the exception of those mixed with monovalent cations such as Na^+^, K^+^, and Li^+^.^[^ ^11^ ^]^ Consequently, studies using pure Ca^2+^‐based electrolytes composed of the above salts and carbonate solvent have only used half‐cells with activated carbon as the counter electrode to examine the potential cathode materials; notably, full‐cell tests with Ca metal anodes have not been conducted.^[^ ^12^ ^]^

In 2019, the research groups of Zhao‐Karger and Nazar simultaneously demonstrated reversible Ca plating/stripping at room temperature and reported high anodic stability (>4 V vs Ca^2+^/Ca) using a fluorine‐type electrolyte (possessing fluorine‐terminated anions) Ca[B(hfip)_4_]_2_ (hfip = hexafluoroisopropyloxy) in an ether solvent.^[^ ^13^ ^]^ Noteworthy progress has been made using this advanced electrolyte in full cells with Ca metal anodes and several potential cathodes.^[^ ^14^ ^]^ However, this electrolyte causes severe passivation on Ca metal anodes and increases the overpotential,^[^ ^15^ ^]^ resulting in inferior cycling stability in terms of the full‐cell performance.^[^ ^16^ ^]^ Instead of Ca metal anodes, alloy‐type anodes such as CaSe*_x_* and CaSn*_x_* have recently been examined;^[^ ^17^ ^]^ for instance, Zhao‐Karger et al. developed a full cell using a novel alloy‐electrode that exhibited extremely high cycling performance over 5000 cycles.^[^ ^16a^ ^]^

Hydride‐type electrolytes (possessing hydrogen‐terminated anions) are another potential electrolyte system that is intrinsically stable against electrodes based on metals, such as Li,^[^ ^18^ ^]^ Na,^[^ ^19^ ^]^ Mg,^[^ ^20^ ^]^ Zn,^[^ ^21^ ^]^ and Ca,^[^ ^22^ ^]^ because of their high reductive stability.^[^ ^23^ ^]^ Wang et al. employed Ca(BH_4_)_2_ in an ether solvent, which exhibited highly efficient Ca plating/stripping behavior without remarkable passivation.^[^ ^24^ ^]^ Using a mixed electrolyte containing Ca(BH_4_)_2_ and LiBH_4_, Jie et al. developed a full cell based on Ca/lithium titanium oxide (LTO) that operated in a voltage window of 1.8–0.8 V and exhibited long‐term stability for 200 cycles.^[^ ^25^ ^]^ In addition, Meng et al. further developed a full cell using a FeS_2_ cathode and a Ca metal anode that exhibited a capacity of 303 mAh g^−1^ at 200 cycles.^[^ ^26^ ^]^ Although these electrolytes were compatible with Ca metal, the voltage window in the oxidative direction was as narrow as 2.4 V versus Ca^2+^/Ca because of the reducing nature of BH_4_ anions.^[^ ^27^ ^]^ A new hydride‐type electrolyte—Ca(CB_11_H_12_)_2_ in dimethoxyethane/tetrahydrofuran (DME/THF)—was recently shown to exhibit a wide electrochemical potential window (up to 4 V vs Ca^2+^/Ca) and high conductivity (4 mS cm^−1^), in addition to supporting reversible Ca metal plating/stripping at room temperature.^[^ ^22^ ^]^ In a feasibility study, a Ca–S battery with this electrolyte exhibited reversible discharge/charge abilities, as well as high capacity (805 mAh g^−1^). However, the long‐term operational characteristics of batteries with Ca(CB_11_H_12_)_2_ in a DME/THF electrolyte have not been reported.

To this end, this study focused on a CuS cathode material that enables the storage of various cations such as Li^+^,^[^ ^28^ ^]^ Na^+^,^[^ ^29^ ^]^ Mg^2+^,^[^ ^30^ ^]^ and Zn^2+^.^[^ ^31^ ^]^ Leon et al. operated a Ca–CuS battery using the fluorine‐containing anion electrolyte Ca tetrakis(perfluoro‐tert‐butoxy) aluminate (Ca[TPFA]_2_) in DME.^[^ ^32^ ^]^ However, this battery exhibited low capacity (90 mAh g^−1^) in the first cycle at a low current density (56 mA g^−1^), and a significant increase in the overpotential above 1.5 V during four‐cycle battery operation. The hindered battery performance was probably caused by the suboptimal electrode design, such as the CuS particle size and the composite structure using carbon materials, and the inadequate electrolyte function at the Ca metal/electrolyte interface, such as the low Coulombic efficiency (CE) of ≈50% for the Ca metal plating/stripping.

In this study, the long‐term operation of Ca metal batteries with a CuS nanoparticle/carbon composite cathode and a hydride‐based electrolyte (Ca(CB_11_H_12_)_2_ in DME/THF) was achieved. The nanoparticles in the carbon composite were highly dispersed in the high‐surface‐area carbon, thereby preventing the aggregation of CuS particles and degradation of the composite electrode during cycling. Additionally, the hydride‐type electrolyte served as an effective electrolyte with reversible Ca metal plating/stripping (CE = ≈90%) and a wide voltage window (>4 V). The assembled Ca–CuS battery exhibited high capacity (390 mAh g^−1^) at 50 mA g^−1^ current density. Ex situ X‐ray photoelectron spectroscopy (XPS) and X‐ray diffractometry (XRD) performed before and after the battery tests revealed changes in the valence state of Cu and amorphization of CuS during battery operation. Furthermore, extremely stable cycling performance was achieved, with 92% capacity retention over 500 cycles (based on the capacity at the 10th cycle). This study demonstrates the feasibility of long‐term Ca metal battery operation using a pure‐Ca^2+^ electrolyte containing a hydride‐based anion.

## 2 Results and Discussion

### 2.1 Characterization of the CuS/C Composite

To prepare a composite in which CuS nanoparticles were highly dispersed in a carbon material, hollow‐structured carbon black (Ketjen Black, KB; EC600JD, Ketjen Black International Company) with a high specific surface area (SSA) of 1270 m^2^ g^−1^ was selected as the carbon matrix. In situ syntheses of CuS and its nanocomposite with the carbon matrix were performed using a hydrothermal method (see detailed synthesis procedure in the Experimental Section).^[^ ^33^ ^]^

**Figure 1:**
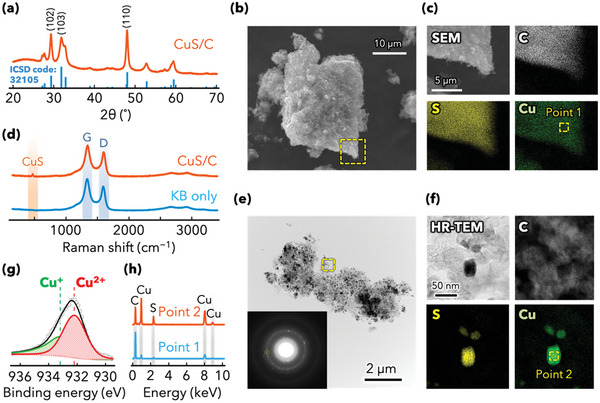
Structural characterization of the CuS/C composite. a) XRD pattern of the CuS/C composite. The major diffraction peaks ((110), (102), and (103)) are indexed to CuS with the Pnma space group (ICSD code no. 32 105). The broad peak at ≈25° corresponds to the (002) plane of KB. b,c) Low‐ and high‐magnification SEM images of the CuS/C composite and EDS maps of C, S, and Cu. d) Raman spectra of CuS/C and KB. e,f) Low‐ and high‐magnification HRTEM images of the CuS/C composite, the corresponding SAED pattern (inset), and EDS maps of C, S, and Cu. g) High‐resolution Cu 2p XPS profile of CuS/C deconvoluted into Cu^2+^ and Cu^+^. h) EDS profiles of CuS/C within the yellow squares for Points 1 and 2 in (b) and (e), respectively.

**Figure** **1**a and Figure S1, Supporting Information, show the XRD patterns of the synthesized CuS/C composite and commercial CuS materials. All diffraction peaks of the prepared and commercial CuS (denoted as CuS‐Aldrich) were indexed and assigned to the hexagonal CuS phase with the space group P6_3_/mmc (JCPDS card no. 06–0464). The broader diffraction peaks of the CuS/C composite compared to those of CuS‐Aldrich indicated a markedly reduced grain size. Assuming that the half‐bandwidth of the XRD peaks depends on the crystal size, the crystallite sizes were calculated using the Scherrer equation for the three planes. The calculated diameter of the CuS crystallites was 10–20 nm, indicating the successful synthesis of the CuS nanoparticles in the carbon matrix. In the XRD pattern of the CuS/C composite, a relatively high peak intensity of the (102) plane was observed because of the in situ synthesis with the carbon nanocomposite. This result suggests that the CuS nanoparticles in CuS/C could have induced the crystalline orientation of the (102) plane, resulting in the high‐surface‐area oriented particles exhibiting high reactivity toward the Ca^2+^ cations. Figure 1d shows the Raman spectra of the CuS/C composite and KB. The intensity ratios associated with the D and G bands (*I*_D_/*I*_G_) are 1.10 (KB) and 1.12 (KB), respectively, indicating that the obtained composite inherits the features of KB. In addition to the carbon peaks, a vibrational mode is observed at 475 cm^−1^, which is typically attributed to stretching modes from the covalent S—S bonds in hexagonally structured CuS.^[^ ^34^ ^]^

The structure of the composite, dispersibility of CuS in the carbon matrix, and aggregability of the CuS particles were examined using scanning electron microscopy (SEM), high‐resolution transmission electron microscopy (HRTEM), and energy‐dispersive X‐ray spectroscopy (EDS); the results are shown in Figure 1b,c,e,f. The aggregated CuS/C composite shows a cotton‐like structure with an average size of 5–30 µm, which is more similar to that of pristine KB (Figure S2, Supporting Information) than that of commercial CuS particles (Figure S3, Supporting Information). EDS results (yellow box shown in Figure 1b) confirm the existence of Cu and S derived from CuS, in addition to C and O from graphitic carbon and the functional groups of KB. The distributions of all the elements (C, Cu, and S) overlap in the entire area, suggesting that the CuS particles in the composite are adequately dispersed in the KB matrix and are entangled.

To comprehensively examine the nanoscale structure of the composite, HRTEM was performed using a dispersion of the CuS/C composite in an ethanol solution (Figure 1e,f). Images of the composite exhibit dark‐colored particles dispersed in a light‐colored matrix, which is presumably KB. In the main sample region, nanoparticles with sizes of 10–50 nm are found in the space between the graphitic carbon particles. In addition to the uniformly dispersed composite, large plate‐like CuS particles (>50 nm) are also found in the space between the graphitic carbon particles (Figure S4, Supporting Information). Although present at levels below 1%, they could affect the electrochemical performance. The selected area electron diffraction (SAED) pattern shows dotted rings corresponding to the (102), (103), and (110) planes of CuS, and rings corresponding to the (101) and (002) planes of KB (Figure 1e, inset). High‐magnification EDS mapping profiles reveal that the dark‐colored particles contain Cu and S rather than C, indicating that these particles represented CuS. The XPS analysis of the CuS/C composite (Figure 1g) shows that the Cu 2p spectrum can be deconvoluted into two peaks representing Cu^+^ and Cu^2+^, which suggests that CuS particles are partially reduced by the solvent (ethanol).^[^ ^31^, ^35^ ^]^ The Cu^+^/Cu^2+^ peak area ratio (0.36/0.64) corresponds to a Cu oxidation state of +1.64 and a copper sulfide composition of CuS_0.82_ (assuming that sulfur is present as S^2−^). The EDS profile of Point 2 shows higher Cu and S peak intensities than those in the profile of Point 1 (Figure 1h), indicating that the CuS particles are highly dispersed in the KB matrix, at least on the scale of 1 µm. These highly dispersed entangled structures can provide electron‐transport pathways to each CuS particle from the current collector through the carbon, and they prevent the aggregation of CuS particles during the charge/discharge processes occurring with the conversion reaction, which typically induces volume expansion and aggregation thereafter.

### 2.2 Ca‐Storage‐Based Electrochemical Characterization of CuS/C

**Figure 2:**
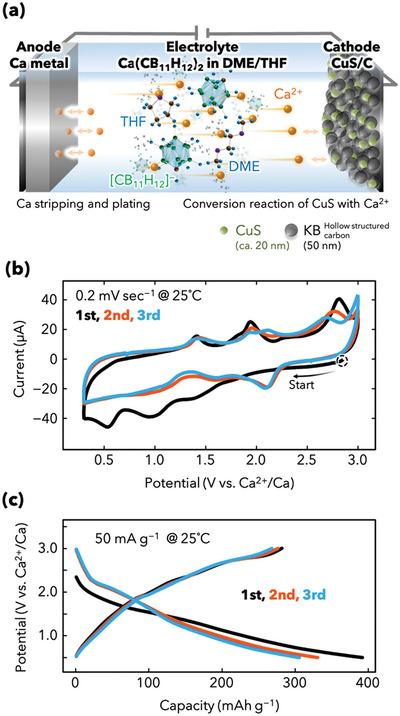
Ca storage performance of the CuS positive electrode. a) Schematic of a prototype Ca metal battery. The battery comprises a Ca^2+^‐storing positive electrode containing the CuS/C composite and a Ca plating/stripping negative electrode with a hydride‐based electrolyte, Ca(CB_11_H_12_)_2_ in DME/THF. b) CV curves of the first three cycles obtained at a scan rate of 0.2 mV s^−1^ in a potential window of 0.3–3 V versus Ca^2+^/Ca. c) Discharge/charge profiles of the first three cycles acquired at a current density of 50 mA g^−1^ in a voltage window of 3–0.5 V at room temperature (≈25 °C).

The Ca storage properties of the CuS/C composite cathode operated with a Ca metal anode as the counter electrode were evaluated using the electrolyte Ca(CB_11_H_12_)_2_ in DME/THF, which is one of the few candidate electrolytes for Ca metal batteries at present (**Figure** **2**a). Electrochemical characterization was performed at room temperature (≈25 °C). Ca(CB_11_H_12_)_2_ in DME/THF contains weakly coordinating [CB_11_H_12_]^−^ anions, which can facilitate Ca metal plating/stripping at room temperature with anodic stability of >4 V.^[^ ^22^ ^]^ The cyclic voltammetry (CV) profile of a Ca/Au cell configuration using Ca(CB_11_H_12_)_2_ in DME/THF shows a reversible reductive/oxidative current corresponding to Ca plating/stripping behavior, whereas no current is generated at 0 V by the cell with Ca(TFSI)_2_ in DME/THF, in which Ca cations and TFSI anions exhibit strong interactions that resulted in limited‐to‐no plating/stripping behavior (Figure S5, Supporting Information). To permit the utilization of Ca metal electrodes for long‐term operation, this study used a pure‐Ca^2+^ electrolyte because an alien cation additive can alter the chemistry and morphology of the original metal electrode.^[^ ^36^ ^]^

The CuS/C composite was applied onto an Al current collector and investigated using CV in a voltage window of 0.3–3 V with a three‐electrode cell setup, which can accurately measure the potential of the working electrode (CuS/C composite electrode) to eliminate the influence of the other electrode (Ca metal electrode). Figure 2b shows the initial three CV curves acquired at 0.2 mV s^−1^. In the first cycle, three broad irreversible reduction peaks appear below 1.7 V, which correspond to the reaction between Ca^2+^ and CuS/C and the formation of a cathode electrolyte interphase (CEI) layer via the partial decomposition of the ether electrolyte. The profile at the oxidation side exhibits oxidation peaks at ≈1.4, 2, and 2.7 V versus Ca ^2+^/Ca, which are mostly reversible with minor peak changes at 2 V in relation to those for the subsequent cycles. Moreover, the CV curves for the second and third cycles show reduction peaks at ≈2.1, 1.8, and 1.1 V. The CV profile of the reduction side of CuS/C for the first cycle differs considerably from those of the second and third cycles, suggesting irreversible crystal transformation from CuS to other phases. These reduction and oxidation peaks indicate that the entire conversion reaction is associated with the emergence of at least three transformations. A comparison of the CV profiles obtained using other inserted cations such as Li^+^, Mg^2+^, and Zn^2+^ reveals that Ca^2+^ storage in CuS involves a multistep reaction, similar to the reaction of Na^+^ with CuS.^[^ ^28^, ^29^, ^31^, ^34^ ^]^ CuS is a unique material whose reaction mechanism with cations largely depends on the cation size. Comparing the storage of monovalent cations, the Na^+^ storage reaction in CuS shows a multistep slope discharge/charge profile, whereas the Li^+^ storage reaction shows a two‐step plateau‐like profile. This multistep slope profile was explained by the large ionic and atomic radii of Na^+^, which introduced a large local strain into the host lattice.^[^ ^37^ ^]^ The large strain promotes the crystallographic tuning of the structure and thus generates a reaction pathway that deviates from the thermodynamic equilibrium. The Na^+^ storage reaction involves the transformations of several distinct phases. Furthermore, a thermodynamically unfavorable phase of Na_7_(Cu_6_S_5_)_2_ is generated as a structural bridge between preceding and following stable phases. This complex reaction mechanism is reflected in the multistep slope discharge/charge profile of the Na‐CuS system. We incorporated this finding when determining the divalent cation storage reaction in CuS. The storage reactions of Mg^2+^ (7.2 Å) and Zn^2+^ (7.4 Å), whose cation sizes are close to that of Li^+^ (7.6 Å), in CuS also show two‐step plateau‐like profiles. However, the storage reaction of Ca^2+^ (10 Å), which is more similar in size to Na^+^ (10.2 Å), shows a multistep slope discharge/charge profile. The differences in the discharge/charge profiles of the differently sized cations Mg^2+^, Zn^2+^, and Ca^2+^ indicate that the reaction mechanism between CuS and divalent cations also depends on the cation size, similar to the reaction with monovalent cations.

Figure 2c shows the first three discharge/charge curves acquired in the voltage range of 0.5–3 V at a current density of 50 mA g^−1^. The curves corresponding to the first discharge/charge process exhibit high capacities (390 and 290 mAh g^−1^), whereas those for CuS‐Aldrich exhibit low capacities (7 and 6 mAh g^−1^, Figure S6, Supporting Information). This indicates that the highly reversible capacities in the reaction between Ca^2+^ and CuS are strongly related to the carbon composite and nanoparticulation of CuS. The profiles corresponding to the second and third cycles almost overlap, which is consistent with the CV results. The mechanism of the reaction between CuS and Ca^2+^ and the contribution of the capacity from carbon materials (KB) are described in later sections.

### 2.3 Mechanism of Ca Storage in CuS

**Figure 3:**
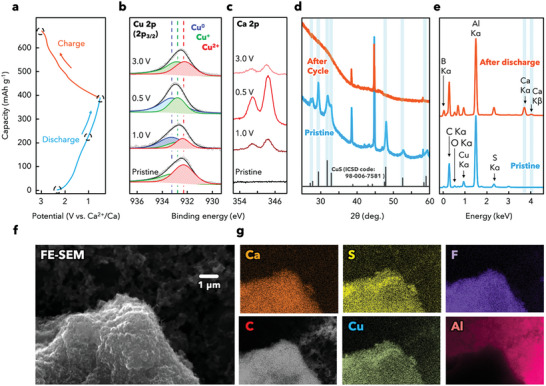
Ex situ analyses of the Ca storage mechanism in CuS. a) Galvanostatic discharge/charge curve of the CuS/C positive electrode acquired during the first cycle at 100 mA g^−1^. b,c) High‐resolution b) Cu 2p and c) Ca 2p XPS profiles of CuS/C electrodes in different electrochemical states: as‐prepared, first discharge to 1 V, first discharge to 0.5 V, and first recharge to 3 V. d) Ex situ XRD patterns of CuS/C before and after the first cycle. e) EDS profiles acquired before and after the first discharge. f) SEM image of the CuS/C composite. g) EDS maps of Ca, S, C, Cu, F, and Al acquired in the fully discharged state.

The mechanism of the reaction between CuS and Ca^2+^ was investigated by conducting ex situ XPS, XRD, and SEM/EDS. XPS was performed using the pristine CuS/C electrode in an air‐free atmosphere at 1 V in the first discharge, 0.5 V in the fully discharged state, and 3 V in the fully charged state; additionally, the discharge/charge curve was acquired (**Figure** **3**a). The XPS profiles (Figure 3b) indicate that the Cu species are present in the form of Cu^2+^, Cu^+^, and Cu^0^, which is confirmed by the observation of peaks at 932.2, 932.7, and 933.2 eV, respectively. The Cu 2p spectrum of the pristine electrode is deconvoluted into two fitted peaks representing Cu^+^ and Cu^2+^, and no Ca 2p peak appears (Figure 3c). Upon discharging to 1 V, the Cu 2p spectrum shifts to higher binding energies and shows three peaks corresponding to Cu^2+^, Cu^+^, and Cu^0^, with the peak for Cu^0^ appearing and that for Cu^2+^ decreasing in intensity, indicating the transformation of CuS to Cu_2_S and subsequently to Cu. The Cu^+^ peak almost maintains its intensity, presumably because of the mutual compensation of the multistep conversion reaction. In the fully discharged state at 0.5 V, the Cu 2p spectrum further shifts to higher binding energies and the Cu^2+^ peak disappears, indicating the phase transformation of CuS to Cu_2_S and then to Cu. The spectrum of the subsequent recharged state at 3 V is deconvoluted into two peaks representing Cu^2+^ and Cu^+^, suggesting successful reversible cycling. Furthermore, the peak for Cu^0^ appeared again in the spectrum of the discharged state at 0.5 V, as shown in Figure S7, Supporting Information. As the discharge/charge process progresses, the changes in the intensity of the Ca 2p peaks are confirmed to be consistent with the variations in the valence states shown in the Cu 2p spectra (Figure 3b). The leftover Ca 2p peak in the fully charged state corresponds to partially unreacted residual CaS or CEI components including Ca(CB_11_H_12_)_2_ salts. To investigate the CEI, we additionally performed XPS on C and O before and after the discharge test, which confirmed that the O peak shifted, while the C peak remained unchanged (Figure S8, Supporting Information). The deconvoluted O peaks also showed an increase in the ether‐type state (—O—C—O—), confirming the presence of polymeric CEI derived from the ether solvents, DME and THF. In addition, HRTEM observation and EDS analysis of the CuS/C electrode after discharge and charge tests confirmed that some Ca was distributed in the electrode, even after charging to remove Ca, which was consistent with the Ca 2p XPS profile (Figure 3c). Furthermore, the EDS map shows that Ca was not distributed only around the CuS particles but throughout the composite region, indicating that decomposition products consisting of Ca formed throughout the composite structure (Figure S9, Supporting Information). These analyses indicate that CEI is a polymeric material derived from ether solvent and Ca(CB_11_H_12_)_2_ salt, which thinly covers the composite surface.

After the first discharge/charge process, the crystal structure of CuS completely disappears and only the pattern of the Al current collector remains. The disappearance of the CuS peaks indicates the amorphization of CuS during its conversion reaction with Ca^2+^. In general, conversion reactions with nanoparticles, such as CuS,^[^ ^38^ ^]^ Mn_3_O_4_,^[^ ^39^ ^]^ SnO_2_,^[^ ^40^ ^]^ and CoO,^[^ ^41^ ^]^ lead to amorphization or further nanoparticulation, which prevents the collection of diffraction patterns. These phenomena occur presumably because the CuS nanoparticles in the CuS/C composite have an average particle size of ≈20 nm. Based on the changes in the valence state of Cu in CuS/C calculated by the deconvolution of the XPS profiles, the reaction could be formulated as the following equations

(1) $$\begin{array}{ccc} & & \mathrm{Discharge}:\mathrm{Cu}S_{0.82}+0.64{\mathrm{Ca}}^{2+}\\ & & \;\to 0.64\mathrm{Cu}+0.18{\mathrm{Cu}}_{2}S+0.64\mathrm{CaS} \end{array}$$

(2) $$\begin{array}{ccc} & & \mathrm{Charge}:0.64\mathrm{Cu}+0.18{\mathrm{Cu}}_{2}S+0.64\mathrm{CaS}\\ & & \;\to {\mathrm{CuS}}_{0.65}+0.47{\mathrm{Ca}}^{2+}+0.17\mathrm{CaS} \end{array}$$

The composition of copper sulfide in the prepared composite was determined beforehand as CuS_0.82_ based on the Cu^+^/Cu^2+^ peak area ratio from Cu 2p spectra of XPS analysis (see characterization section). The capacities calculated using these equations for the first discharge/charge process (first discharge: 357 mAh g^−1^; first charge: 264 mAh g^−1^) are consistent with the electrochemically measured capacities during the first cycling step after eliminating the KB‐related capacities (first discharge: 348 mAh g^−1^; first charge: 256 mAh g^−1^). Figure 3e–g shows the SEM/EDS results for the CuS/C composite electrode before and after the discharge process. The EDS profile obtained after full discharge shows peaks related to Ca and B in addition to those corresponding to Cu and S from CuS, Al from the current collector, and C and O from KB, which are observed in the spectrum of the pristine CuS/C electrode. Moreover, the intensity of the O peak increases. These results indicate that the formation originates from the electrolyte containing the Ca(CB_11_H_12_)_2_ salt and ether solvents, which is consistent with the XPS results. Notably, the peak at 0.7 eV corresponds to the F species from the polyvinylidene fluoride binder that was dispersed throughout the entire electrode. Therefore, the intensity of the F peak varies with the investigated location. In terms of morphology, the electrode after the discharge process has a smoother surface than its pristine equivalent (Figure 1b), which is partly derived from the thin CEI layer formed on the composites. Ca is densely distributed across the entire composite, suggesting that Ca^2+^ storage occurred uniformly with the CuS particles dispersed in the KB matrix.

### 2.4 Performance of the Ca Metal Battery

**Figure 4:**
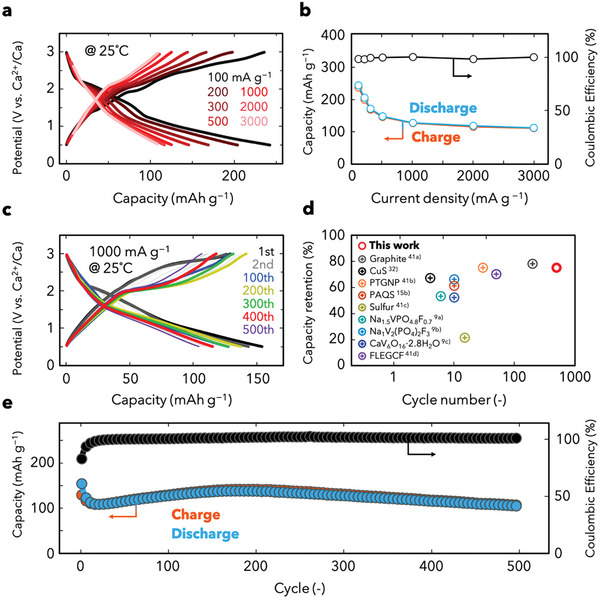
Performance of a Ca metal battery with the CuS positive electrode. a) Galvanostatic discharge/charge profiles acquired at various current densities (100–3000 mA g^−1^). b) Rate capability test results. c) Galvanostatic discharge/charge profiles obtained at 1000 mA g^−1^ for different cycle numbers. d) Cycling performance of previously reported Ca metal batteries with single Ca^2+^ electrolytes; the cycling stabilities for different cycle numbers were analyzed on the basis of their charge/discharge curves.^[^ ^9^, ^16^, ^32^, ^43^ ^]^ e) Cycling performance of the Ca–CuS battery analyzed at 25 °C at a current density of 1000 mA g^−1^.

Rate capability tests of Ca^2+^ storage in the CuS/C composite were performed to evaluate its kinetic performance. **Figure** **4**a shows the typical charge/discharge profiles acquired at various current densities (100–3000 mA g^−1^) after three pre‐cycles performed at 100 mA g^−1^ (Figure S10, Supporting Information). The CuS/C composite exhibits reversible discharge capacities of ≈242, 204, 170, 147, 126, 117, and 110 mAh g^−1^ at 100, 200, 300, 500, 1000, 2000, and 3000 mA g^−1^, respectively (Figure 4b). Two‐step capacities are clearly observed in all the profiles, confirming the conversion reaction of CuS at different current densities. The capacity retentions are relatively stable at current densities above 500 mA g^−1^; 87% (1000 mA g^−1^), 79% (2000 mA g^−1^), and 76% (3000 mA g^−1^) of the capacity at 500 mA g^−1^ are achieved, which are attributed to the composite structure that provides smooth electron‐transport pathways and a Ca^2+^ reservoir to the CuS nanoparticles embedded in KB. The Ragone plot constructed on the basis of the weight of the cathode (Figure S11, Supporting Information) shows that an impressive energy density of ≈306 Wh kg^−1^ can be achieved at a power density of 131 W kg^−1^. Moreover, the battery can deliver a reasonable energy density of 144 Wh kg^−1^ at a high power density of 4000 W kg^−1^, which is comparable to that of previously reported dual‐cation‐system Ca metal batteries.^[^ ^42^ ^]^

The Ca metal batteries with the CuS/C composite electrode were cycled at 1000 mA g^−1^ to evaluate their long‐term cycling performance (Figure 4c–e and Figure S12, Supporting Information). Compared with the profile for the first cycle, the charge curves obtained after the 100th cycle exhibit increased capacities that plateau in the low‐voltage region; this trend is maintained over 500 cycles. The low‐voltage charging and retention of the discharge voltage suggest a decrease in the overpotential between the discharge and charge, leading to energy efficiency. This decrease in overpotential could be derived from the transformation to a more adequate structure via the reaction with Ca^2+^ from the initial state. An extremely high reversible CE is achieved after ten cycles (≈100%; maximal fluctuation of ±0.5%), indicating high reversibility. The cycling performance in terms of capacity retention of 92% after 500 cycles (based on the capacity at the 10th cycle) is the highest observed among previously reported Ca metal batteries with pure‐Ca‐cation electrolytes (Figure 4d and Table S1, Supporting Information).^[^ ^9^, ^16^, ^32^, ^43^ ^]^

To eliminate the contribution of the carbon material to the reversible capacities, a pure KB electrode was prepared and evaluated as the cathode electrode at 1000 mA g^−1^ for the cycle test and at different current densities for the rate test (Figures S13 and S14, Supporting Information). The KB electrode exhibits discharge capacities of 71 and 44 mAh g^−1^ at the initial and 100th cycles, respectively. Figure S12, Supporting Information, shows the capacities at different cycle numbers for CuS, calculated by subtracting the KB capacity from the CuS/C composite capacity and by taking into account the composite ratio of 6/4 (CuS/C). These capacities are 106 and 98 mAh g^−1^ at the initial and 100th cycles, respectively. While the contribution of KB to the capacity is not negligible, the results show that CuS significantly contributes to the reversible capacity of the Ca metal battery.

Cycling tests were performed using the three‐electrode cell, which accurately yielded the potentials for the cathode and anode simultaneously versus a Ca reference electrode. To acquire data related to the cycle‐number‐based variations in the anodic voltage, the discharge/charge profiles obtained using the three‐electrode cell were converted to those of a two‐electrode cell (Figure S15, Supporting Information). Although the converted profiles for different cycle numbers showed that the discharge curves shifted to lower voltages owing to the overpotential of the Ca anode, their overall shape was similar to that of the original profiles. This suggests that the Ca metal batteries can be operated as full‐cell systems using Ca(CB_11_H_12_)_2_ in DME/THF to achieve a long cycle life with a Ca metal anode. Assuming a two‐electrode system, the cycle performance of CuS/C was recalculated from the discharge/charge profile with a cut‐off voltage of 0.5 V. Figure S15b, Supporting Information, shows discharge capacities of 146 and 92 mAh g^−1^ at the initial and 100th cycles, respectively. These findings highlight the feasibility of the long‐term operation of Ca metal anodes and can drive the development of Ca metal batteries.

## 3 Conclusion

In this study, a long‐cycle‐life Ca metal battery was developed using a CuS/C nanocomposite and a tailored monocarborane electrolyte solution of Ca(CB_11_H_12_)_2_ in DME/THF. CuS nanoparticles with a size of ≈20 nm were highly dispersed in a KB matrix by hydrothermal means and simultaneously composited with high‐surface‐area carbon. The reversible Ca^2+^ storage properties of CuS were evaluated by conducting ex situ XRD, XPS, SEM, and HRTEM analyses. The nanocrystalline CuS transformed into an amorphous material during the conversion reaction between CuS and Ca^2+^, and the multistep reaction profiles were retained following the discharge/charge cycling. Prototype CuS/Ca batteries with the electrolyte containing weakly coordinating anions in a mixed ether solvent exhibited higher performance than state‐of‐the‐art Ca metal batteries in terms of both the rate capability and cycle life (Figure 4d and Table S1, Supporting Information). This study highlights the potential of developing practical batteries using Ca metal. The use of cathodes at voltages >3 V could enable improvements in the energy density of Ca metal batteries and facilitate the use of Ca‐ion batteries as practical alternatives to existing LIBs.

## 4 Experimental Section

The preparation and handling of air‐sensitive materials were conducted under a dry Ar atmosphere using a glovebox and Schlenk techniques.

### Materials

Cu(CH_3_COO)_2_·H_2_O (FUJIFILM Wako Pure Chemical Co., Ltd.) and CH_4_N_2_S (Thiourea, Sigma‐Aldrich) were used as the Cu and S sources, while C_19_H_42_BrN (CTAB, Tokyo Chemical Industry Co., Ltd.) was used to synthesize CuS. Hollow‐structured KB (EC600JD, Ketjen Black International Company) with 50 nm‐diameter primary particles and an SSA of 1270 m^2^ g^−1^ was selected as the carbon matrix because of its high electrical conductivity and SSA. Ultrapure water (Sigma‐Aldrich) was used as the medium for the entire preparation scheme. CsCB_11_H_12_ (Katchem Ltd.) and CaCO_3_ (FUJIFILM Wako Pure Chemical Co., Ltd.) were used for the preparation of the Ca(CB_11_H_12_)_2_ salt, while Ambarlite IR120B (DuPont Water Solutions) was used for ion exchange. The solvents, DME (Sigma‐Aldrich) and THF (Sigma‐Aldrich), were used as received.

### Synthesis of the CuS/C Composite

The simultaneous synthesis of CuS and its composite with carbon materials was carried out using the facial hydrothermal method. Two different solutions, A and B, were prepared. Solution A contained 0.1996 g of Cu(CH_3_COO)_2_·H_2_O dissolved in 6 g of water. Solution B contained 0.1903 g of CH_4_N_2_S dissolved in 6 g of water. A 1 m NaOH solution was added gradually under magnetic stirring into solution A to maintain the pH near 8. Subsequently, solution A and 0.0637 g of KB were vigorously mixed for 30 min after ultrasonication treatment for 30 min to obtain a uniform premixture. Solution B was added dropwise to the above black suspension of solution A and KB under vigorous stirring using a magnetic stirrer at room temperature, following which CTAB (0.075 g) was added to the mixed solution. Finally, the mixed solution was transferred into a 25 mL Teflon‐lined stainless‐steel high‐pressure reaction vessel with a fill factor of ≈60%. The autoclave was sealed and heated at 120 °C in an oven for 12 h. After cooling to room temperature, the blackish suspension was filtered, washed several times in water and ethanol, and then dried at 80 °C in a vacuum for 12 h.

### Physicochemical Characterizations of the CuS/C Composite

The prepared sample was identified by XRD using an X'PERT Pro diffractometer (PANalytical) with Cu K*α* radiation (wavelength *λ* = 1.5406 Å for K_*α*1_ and 1.5444 Å for K_*α*2_). The vibrational modes of complex anions were characterized by Raman spectroscopy (DXR, Thermo Scientific). The valence state of Cu in CuS was evaluated by XPS (PHI5000 VersaProbe II, ULVAC‐PHI, Inc.). The microstructure of CuS/C was observed by SEM (JSM‐7800F, JEOL) with EDS and HRTEM (JEM‐ARM200F, 200 kV, Cold‐FE, JEOR). All the samples after electrochemical testing were sealed in air‐tight sample holders to prevent any exposure to ambient conditions during sample transfer.

### Synthesis of Ca(CB_11_H_12_)_2_ in DME/THF Electrolytes

Ca(CB_11_H_12_)_2_ electrolytes were synthesized via ionic exchange and heat treatment. First, CsCB_11_H_12_ (2.759 g, 10 mmol) was converted into the corresponding acid [H_3_O][CB_11_H_12_] through ion exchange (acidic form of Ambarlite IR120B, 20 mL). Aqueous Ca(CB_11_H_12_)_2_ was prepared by neutralizing [H_3_O][CB_11_H_12_] with excess CaCO_3_ (1.5013 g, 1.5 eq). Solvent removal yielded hydrated Ca(CB_11_H_12_)_2_, which was further dried under vacuum (<8 × 10^−4^ Pa) at 433 K for 10 h to obtain Ca(CB_11_H_12_)_2_. To prepare Ca electrolytes, Ca(CB_11_H_12_)_2_ was dissolved in a volumetric flask with appropriate amounts of a DME/THF mixed solvent to achieve the desired or saturated concentration. The molar concentration of the electrolyte was based on the molar mass of Ca(CB_11_H_12_)_2_.

### Electrochemical Analyses and Battery Tests

Disk‐shaped working electrodes composed of Au with a diameter of 8 mm, and counter and reference electrodes of Ca with diameters of 8 and 5 mm, respectively, were extensively polished until a metallic luster was achieved before each use. All the electrochemical analyses were performed at room temperature with a stainless‐steel electrochemical cell holder (EC‐Frontier). CV was conducted at 20 mV s^−1^ with the voltage ranging between −0.4 and 4 V versus Ca^2+^/Ca using multi‐channel potentiostats (Cell Test System 1470E, Solectron Analytical). CuS/C electrodes were prepared by mixing 80 wt% of the composite and 20 wt% of the weight of polyvinylidene difluoride (PVDF) in *N*‐methyl pyrrolidone. The mixture was coated on an etched Al foil (current collector) and dried at 80 °C in a vacuum for 12 h. For the battery tests, the CuS/C electrodes, separator, electrolyte, and Ca metal anode were placed in a stainless‐steel electrochemical cell holder. The electrochemical measurements were conducted at several current densities at room temperature in the voltage range of 3–0.5 V using a battery test system (580 Battery Test System, Scribner Associates, Inc.)

## Conflict of Interest

The authors declare no conflict of interest.

## Supporting information

Supporting Information

## Data Availability

The data that support the findings of this study are available from the corresponding author upon reasonable request.
