# Supplementary material for: Calcium Metal Batteries with Long Cycle Life Using a Hydride‐Based Electrolyte and Copper Sulfide Electrode
Source: Adv Sci (Weinh). 2023 May 19;10(22):2301178. doi: 10.1002/advs.202301178 (PMC10401151; doi:10.1002/advs.202301178)
Supplement: Supplementary file 1 — Supporting Information [file ADVS-10-2301178-s001.pdf]

ADVANCED SCIENCE
Open Access

Supporting Information

for *Adv. Sci.*, DOI 10.1002/advs.202301178

Calcium Metal Batteries with Long Cycle Life Using a Hydride-Based Electrolyte and Copper Sulfide Electrode

Kazuaki Kisu, Rana Mohtadi and Shin-ichi Orimo**

Supporting Information

Calcium Metal Batteries with Long Cycle Life Using a Hydride-Based Electrolyte and Copper Sulfide Electrode

Kazuaki Kisu, Rana Mohtadi, and Shin-ichi Orimo**

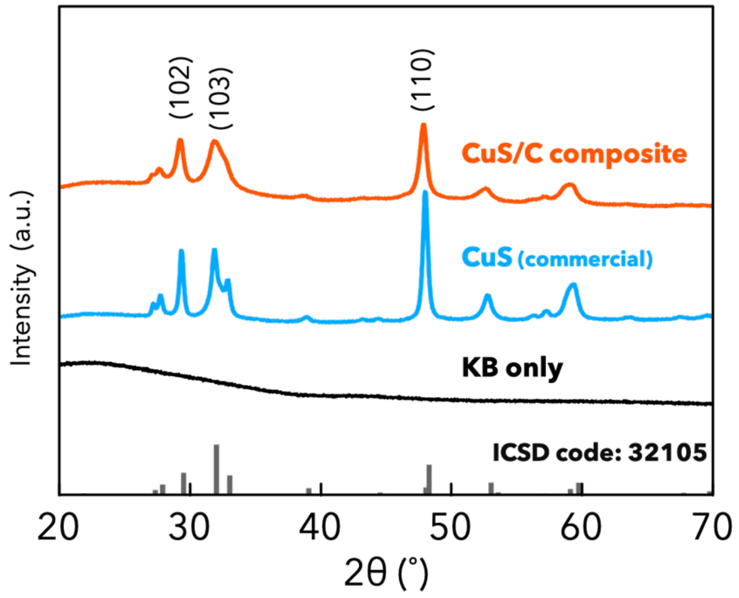

Figure S1. XRD pattern of the CuS/C composite, commercial CuS, and Ketjen Black only. The major diffraction peaks in the pattern of the CuS/C composite and commercial CuS are indexed to CuS with the *Pnma* space group (ICSD code no. 32105).

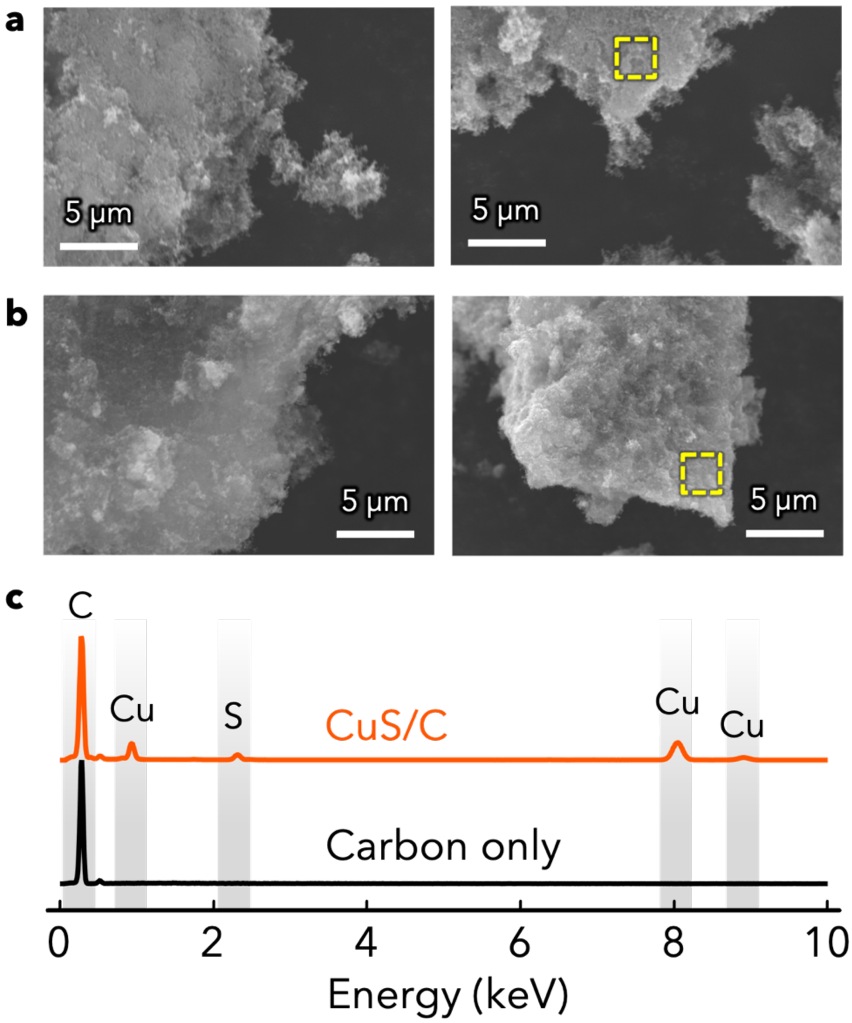

Figure S2. (a, b) SEM images of KB only and the CuS/C composite. (c) EDS profiles of the area within the yellow squares.

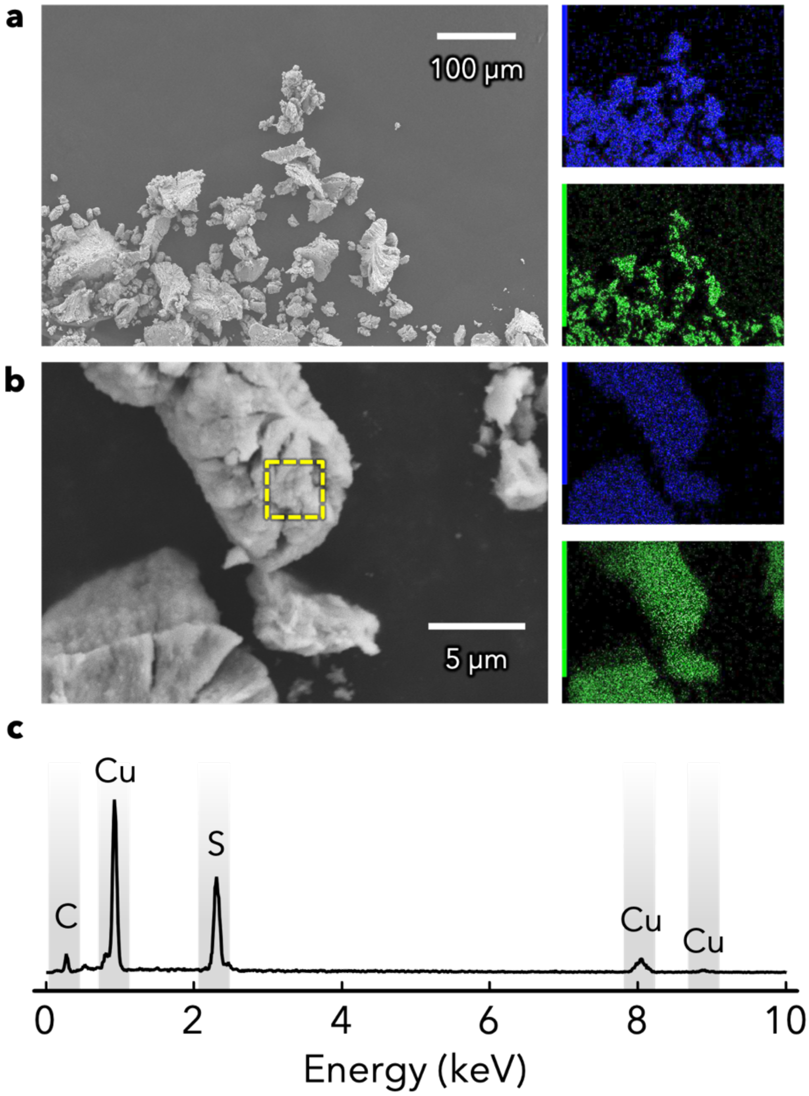

Figure S3. (a, b) Low- and high-magnification SEM images of the commercial CuS particles and EDS maps of Cu and S (top). (c) EDS profiles of commercial CuS within the yellow squares (bottom).

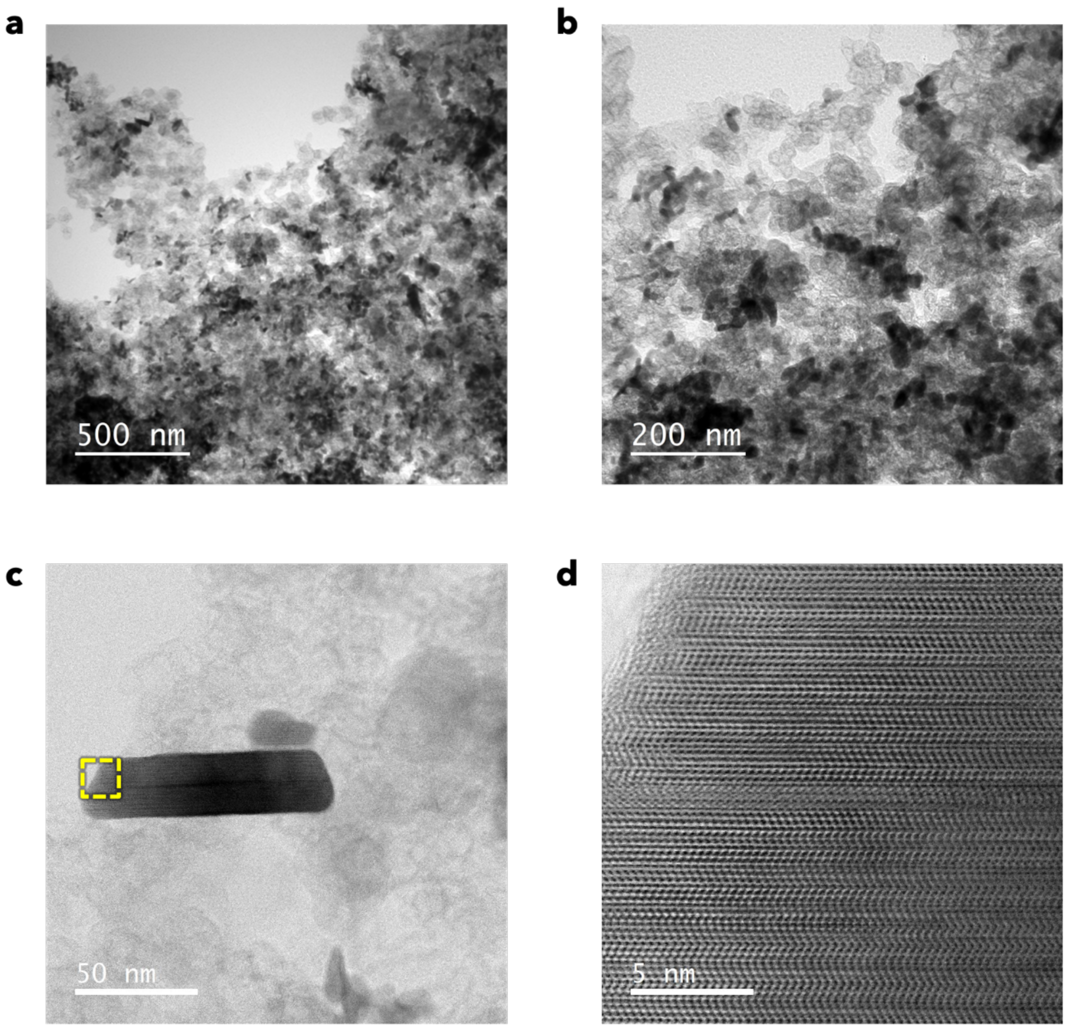

Figure S4. (a-b) HRTEM images for CuS/C composite with different magnification, (c-d) large plate-like CuS particles in composite.

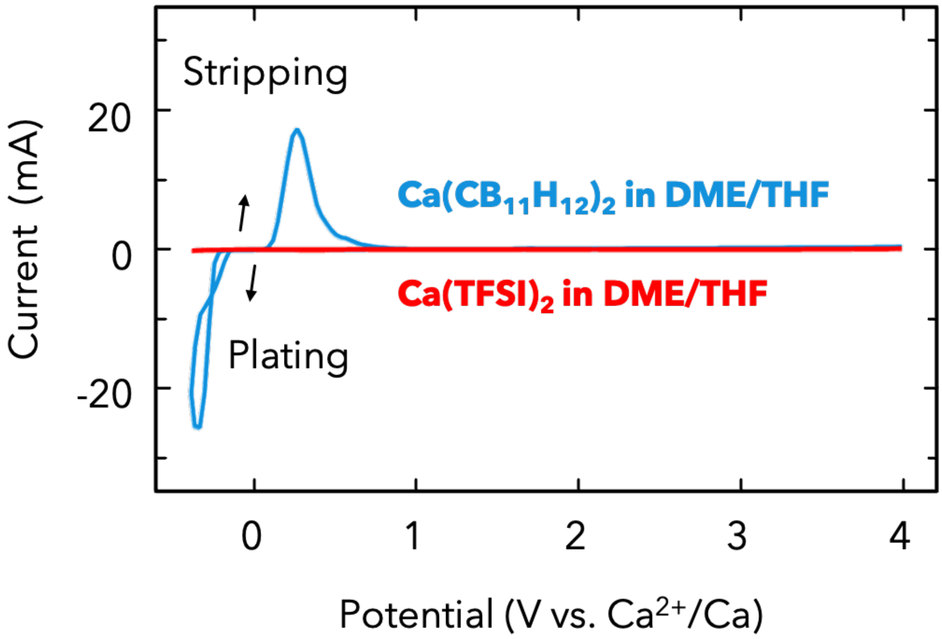

Figure S5. (a) Cyclic voltammogram using different electrolyte of $Ca(CB_{11}H_{12})_2$ in DME/THF (blue) and $Ca(TFSI)_2$ in DME/THF (red) with a Ca metal electrode as the counter and reference electrodes.

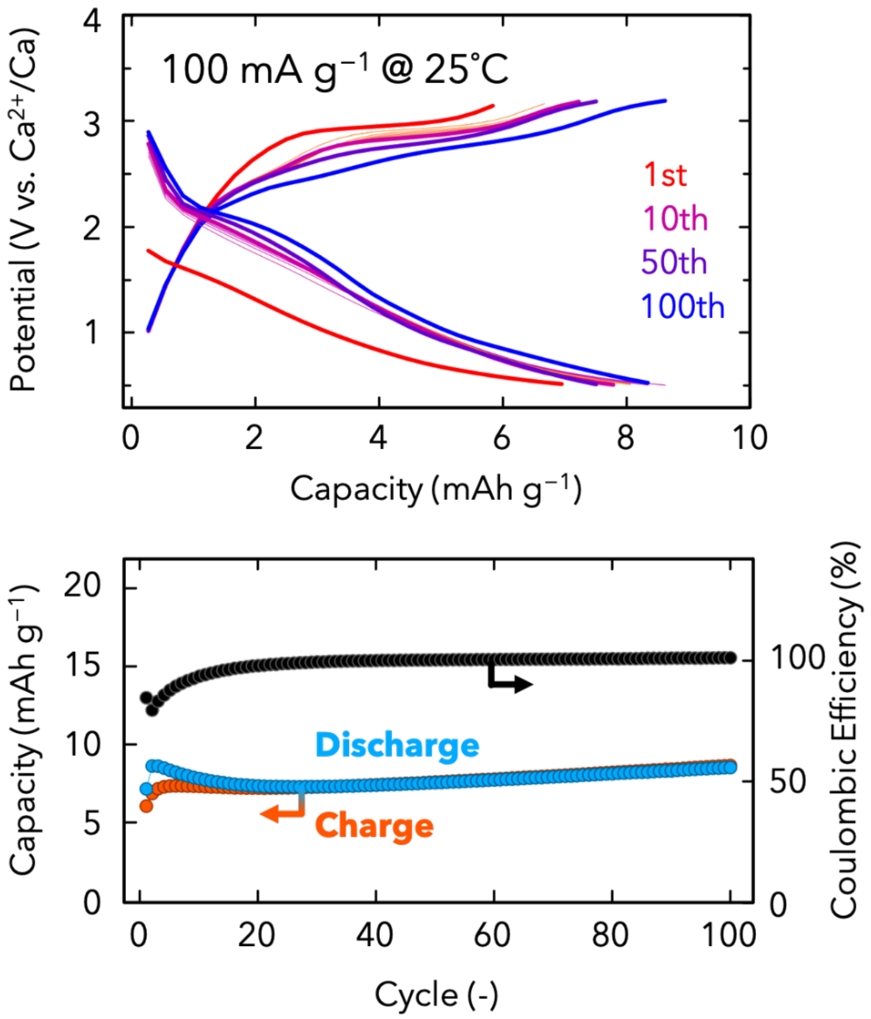

Figure S6. Discharge and charge profiles of commercial CuS powder with AB carbon using $Ca(CB_{11}H_{12})_2$ in a DME/THF electrolyte with a voltage window of 0.5–3.0 V at a current density of 100 mA g^{-1}.

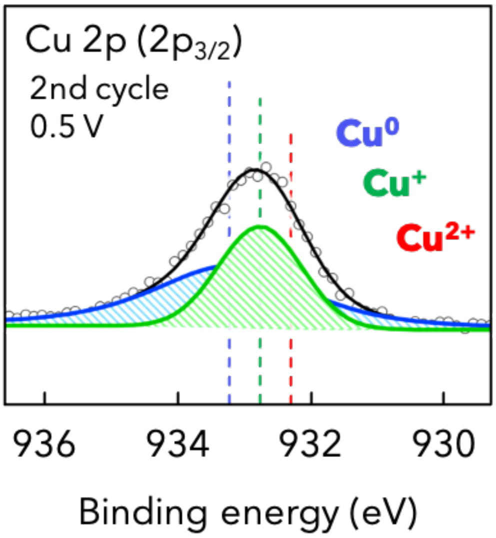

Figure S7. Ex situ Cu 2p XPS profiles of CuS/C electrodes after second discharge to 0.5 V.

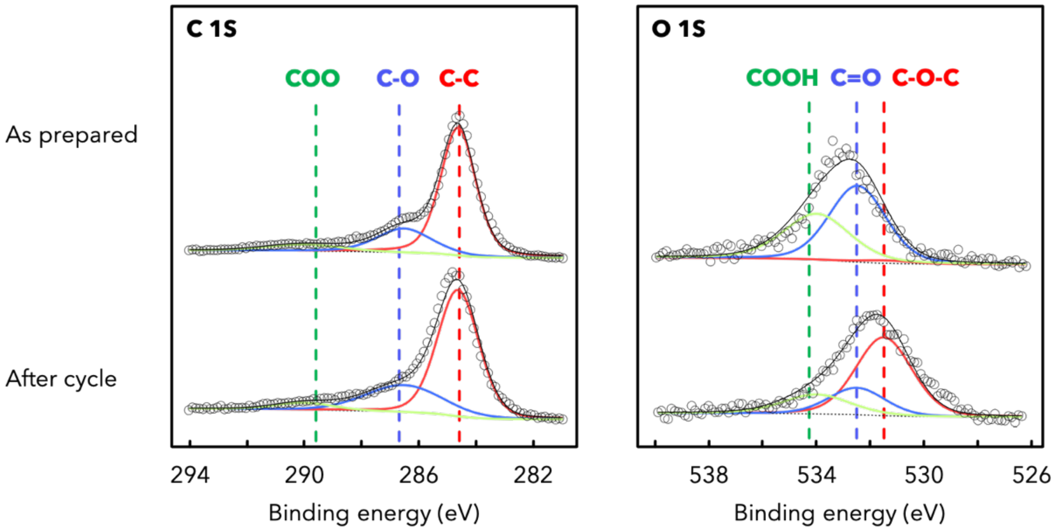

Figure S8. Ex situ C 1s and O 1s XPS profiles of CuS/C electrodes in different electrochemical states: as prepared and first recharge to 3.0 V.

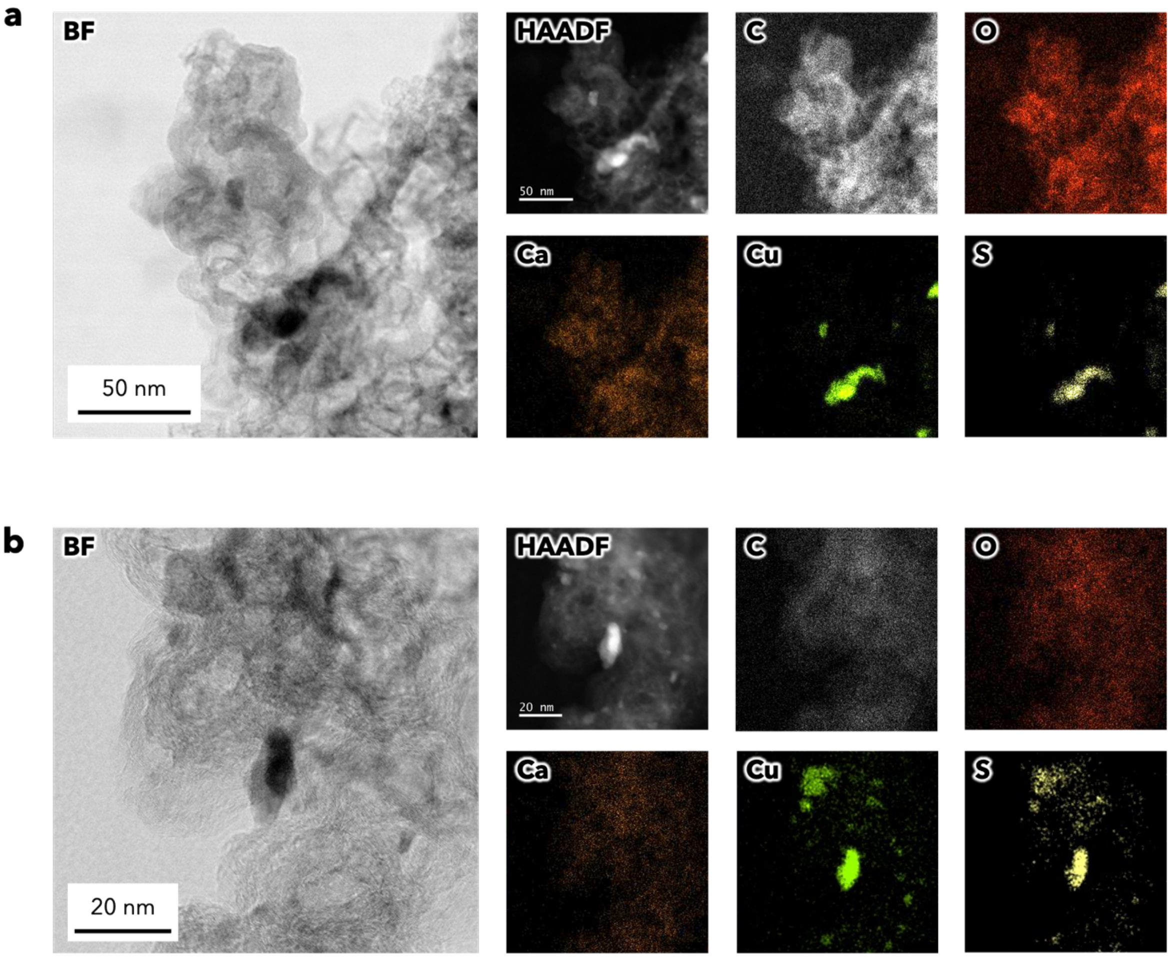

Figure S9. HRTEM images of the CuS/C composite after a discharge and charge test, and EDS maps of C, O, Ca, S, and Cu. Panels (a) and (b) show the same analysis at different points in the composite.

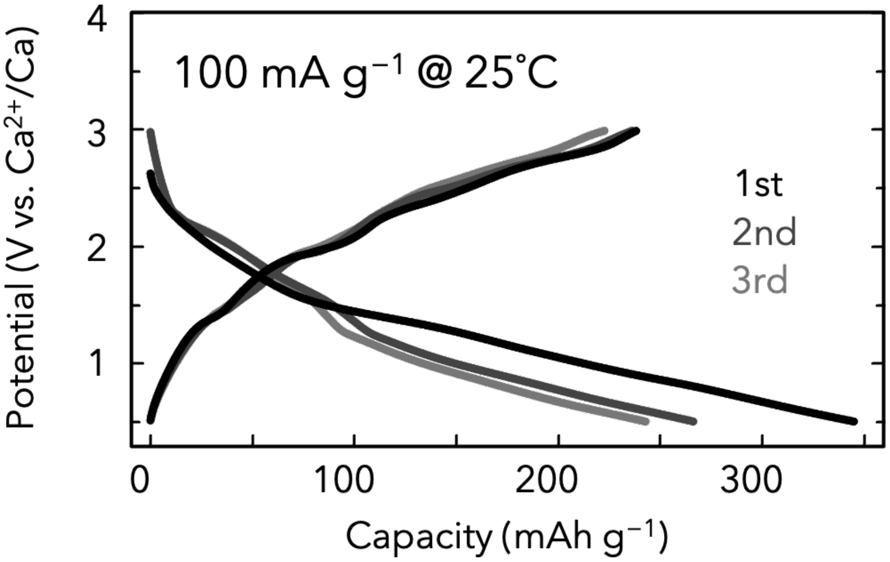

Figure S10. Discharge and charge profiles of CuS/C in the initial three cycles before rate tests using $Ca(CB_{11}H_{12})_2$ in a DME/THF electrolyte with a voltage window of 0.5–3.0 V at a current density of 100 mA g^{-1}.

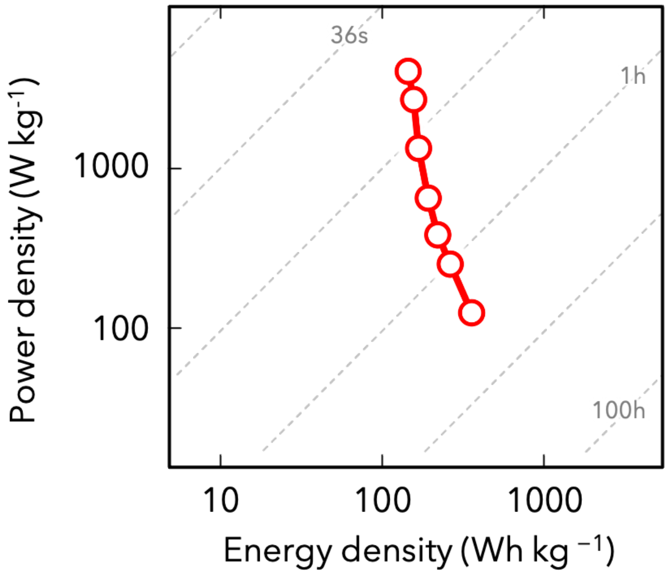

Figure S11. Ragone plots comparing the energy and power performance of Ca metal battery with CuS/C cathode. These plots were prepared from discharge profile data, discharge capacities, and current densities presented in Figure 4a.

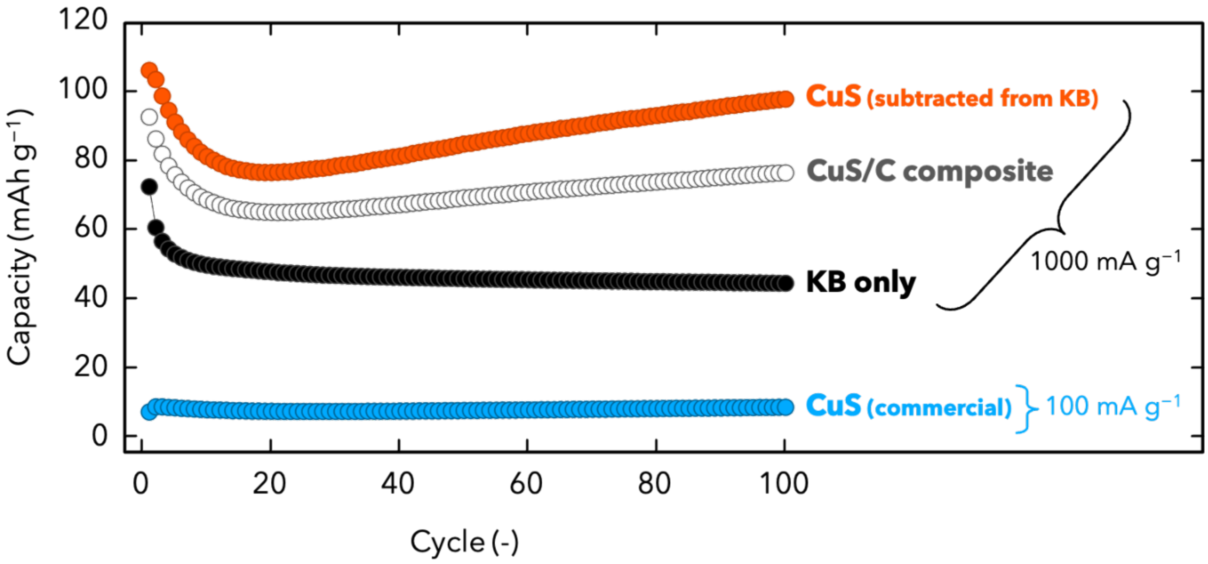

Figure S12. Cycle performances of CuS (orange), CuS/C composite (white), KB only (black), and commercial CuS with AB (blue). The capacity of CuS was calculated by subtracting the capacity of KB from that of the CuS/C composite taking into account the composite ratio of 60/40 (CuS/KB).

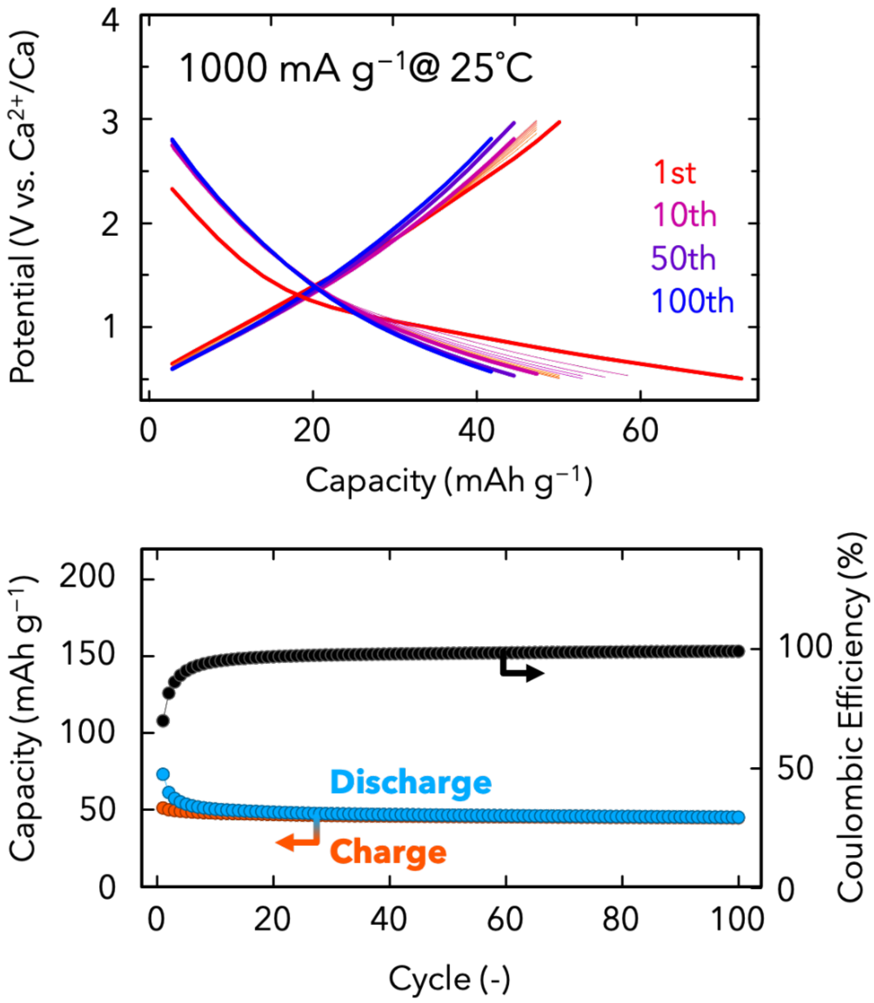

Figure S13. Discharge and charge profiles of KB only with a PVDF binder using $Ca(CB_{11}H_{12})_2$ in a DME/THF electrolyte with a voltage window of 0.5–3.0 V at a current density of 1000 mA g^{-1}.

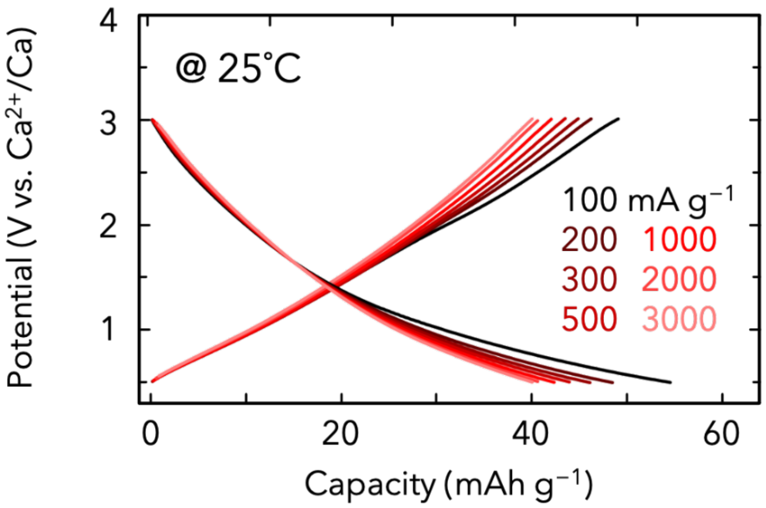

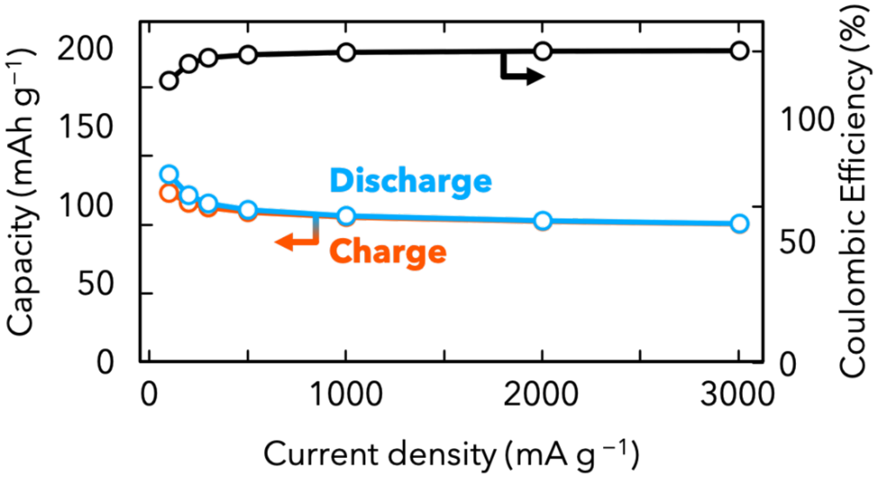

Figure S14. Discharge and charge profiles of KB only with a PVDF binder using $Ca(CB_{11}H_{12})_2$ in a DME/THF electrolyte with different current densities from 100 to 3000 mA g^{-1}.

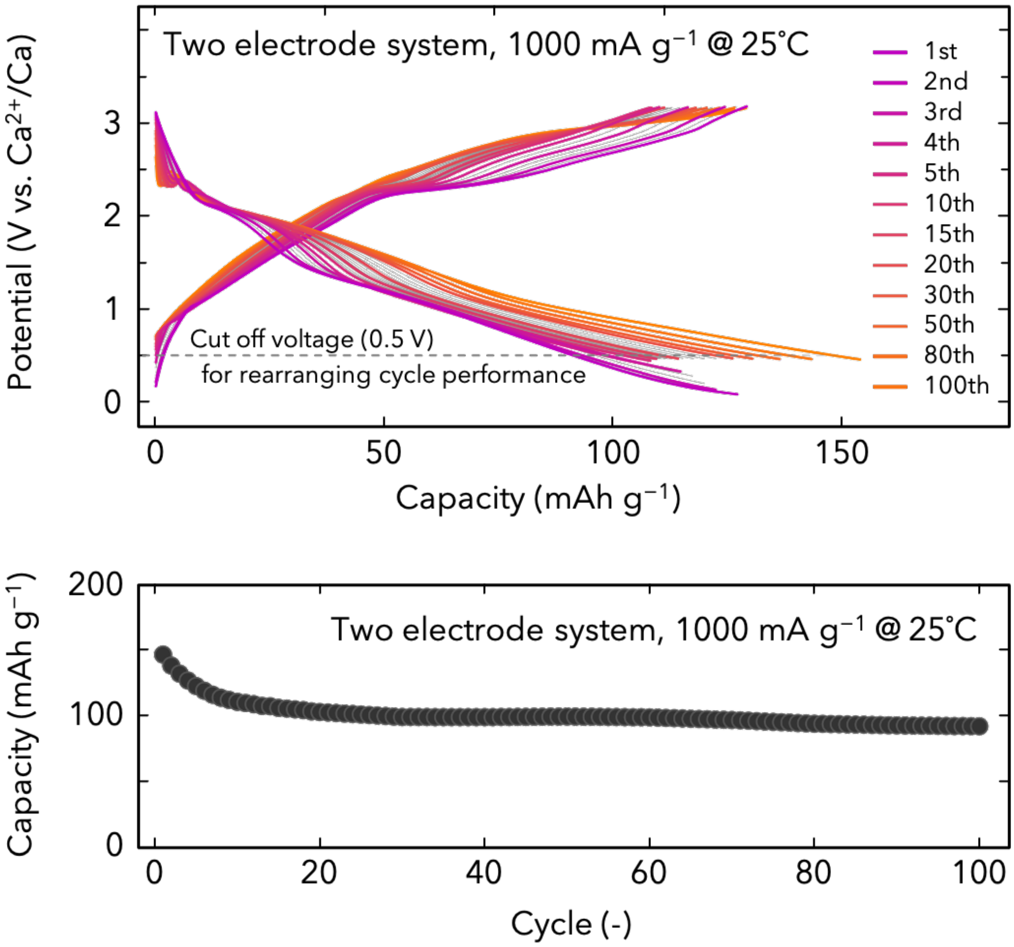

Figure S15. (a) Discharge and charge profiles at 1000 mA g^{-1} with different cycle numbers for a two-electrode cell system, converted from the curves for the working electrode (CuS/C cathode) and counter electrode (Ca metal anode). (b) Cycle performance of CuS/C recalculated for a two-electrode system using the above discharge and charge profiles with a cut-off voltage of 0.5 V.

Table S1. Comparison of state-of-the-art Ca metal batteries. (Dual-cation and hybrid systems are therefore not included.) The calculation for active materials is based on the mass of the cathode. To extract accurate capacity retention values from previous papers, these cycle numbers were selected priority to data obtained from charge-discharge curves were preferentially used in the calculations.[1-11]

Anode	Cathode	1st capacity (mAh g^{-1})	Capacity retention (%)	Cycling stability (cycle number)	Voltage	Ref.
Ca	CuS/C	155	75	500	0.5–3.0 V	This work
Ca	CuS	130	67	4	0.0–3.5 V	[1]
Ca	$CaV_6O_{16}·2.8H_2O$	83.4	52	10	1.0–3.9 V	[2]
Ca	$Na_1V_2(PO_4)_2F_3$	106	66	10	1.5–4.5 V	[3]
Ca	$Na_{0.5}VPO_{4.8}F_{0.7}$	85	53	6	2.0–4.2 V	[4]
Ca	ACC/S	949	21	15	0.5–3.5 V	[5]
Ca	S/C	760	15	15	1.0–3.2 V	[6]
Ca	PAQS/CNTs	188	61	10	1.3–3.3 V	[7]
Ca	Graphite	97	78	200	0.2–1.5 V	[8]
Ca	FLEGCF	89	67	50	1.2–4.5 V	[9]
Ca	14PAQ	253	43	6	1.0–3.0 V	[10]
Ca	PTGNP	60	75	30	2.3–4.1 V	[11]

References

[1] N. J. Leon, X. Xie, M. Yang, D. M. Driscoll, J. G. Connell, S. Kim, T. Seguin, J. T. Vaughey, M. Balasubramanian, K. A. Persson, C. Liao, *J. Phys. Chem. C* **2022**, 126, 13579.
[2] J. Wang, J. Wang, Y. Jiang, F. Xiong, S. Tan, F. Qiao, J. Chen, Q. An, L. Mai, *Adv. Funct. Mater.* **2022**, 32, 2113030.
[3] C. Chen, F. Shi, S. Zhang, Y. Su, Z. L. Xu, *Small* **2022**, 18, e2107853.
[4] Z. L. Xu, J. Park, J. Wang, H. Moon, G. Yoon, J. Lim, Y. J. Ko, S. P. Cho, S. Y. Lee, K. Kang, *Nat. Commun.* **2021**, 12, 3369.
[5] Z. Li, B. P. Vinayan, T. Diemant, R. J. Behm, M. Fichtner, Z. Zhao-Karger, *Small* **2020**, 16, e2001806.
[6] A. Scafuri, R. Berthelot, K. Pirnat, A. Vizintin, J. Bitenc, G. Aquilanti, D. Foix, R. Dedryvère, I. Arčon, R. Dominko, L. Stievano, *Chem. Mater.* **2020**, 32, 8266.
[7] J. Bitenc, A. Scafuri, K. Pirnat, M. Lozinšek, I. Jerman, J. Grdadolnik, B. Fraisse, R. Berthelot, L. Stievano, R. Dominko, *Batter. Supercaps* **2020**, 4, 214.
[8] J. Park, Z. L. Xu, G. Yoon, S. K. Park, J. Wang, H. Hyun, H. Park, J. Lim, Y. J. Ko, Y. S. Yun, K. Kang, *Adv. Mater.* **2020**, 32, e1904411.
[9] H. Song, Y. Li, F. Tian, C. Wang, *Adv. Funct. Mater.* **2022**, 32, 2200004.
[10] Z. Zhao-Karger, Y. Xiu, Z. Li, A. Reupert, T. Smok, M. Fichtner, *Nat. Commun.* **2022**, 13, 3849.
[11] Y. Xiu, A. Mauri, S. Dinda, Y. Pramudya, Z. Ding, T. Diemant, A. Sarkar, L. Wang, Z. Li, W. Wenzel, M. Fichtner, Z. Zhao-Karger, *Angew. Chem. Int. Ed.* **2022**, 6, e202212339.
